# Answers to Querists

**Published:** 1867-10

**Authors:** 


### Answers to Querists.

*Properties, Generation and Administration of Nitrous Oxide Gas.* Query 2nd.—*Generation of Nitrous Oxide Gas? Answer.*

The apparatus required for the generation, purification and preservation of this gas, consists of a *retort*, a *purifier* or *washer*, and a *receiver* or *gasometer*; the three being connected by means of rubber tubing.

The *retort* should be of glass, and to prevent its fracture by the heat, necessary to fuse and boil the nitrate of ammonia, it should be protected by a sand bath consisting of a porcelain or metalic cup partly filled with sand into which the base of the bulb of the retort is buried.

There are several kinds of *purifiers* in use. The *purifier* attached to Snowden and Cowman's Gas Generator, consists of two glass jars, similar in arrangement to what is known as Woulf's apparatus, except that they have no central or safety tubes. One of these jars contains a solution of sulphate of iron; the other a solution of caustic soda or potash, and are so connected by means of the rubber tubing with the retort, in which the gas is generated, and with the receiver or gasometer, that the gas is compelled to pass through the iron and soda solutions, before it can reach the gasometer in which it is stored.

These solutions free the gas from such deleterious agents as chlorine, nitric oxide, hyponitrous acid, &c. Additional care may be taken to free the gas from such agents, by having the gasometer filled with lime water, through which the gas passes in its ascent to the bell of the receiver.

Sprague's *purifier* or washer consists of four glass jars arranged in the same manner as the apparatus just described.

Bean's *purifier* is a cylindrical copper vessel, twelve inches high and five inches in diameter, open at both ends and having a partition of the same material about four inches from the lower end, with a series of holes around the circumference of the cylinder just below this partition.

The upper portion of the cylinder contains several layers of moist lime, separated by intervals, and supported on disks of wire gauze. The whole sits in a vessel of water six inches deep, and is covered with a bell-glass. The pipes are so arranged that the gas passes into the lower portion, bubbling through the holes into the bell-glass, returning downward through the layers of lime, thence through the partition by a pipe leading into the gasometer.

White's *purifier* contains seven inverted glass tumblers which rest upon a perforated metal plate, placed about midway in a vessel. On this plate a number of tubes are soldered, so that two tubes are under each tumbler. The purifier is filled with the required solution a little above the opening of the tubes, and the gas from the retort entering the first tube passes through the second tube into the water contained under the first tumbler; rising to the top of the tumbler it passes into and through another set of tubes contained in the second tumbler, and from thence consecutively under the remaining tumblers, being washed seven times; thence entering by a connecting tube through a pipe (which is provided with a stop-cock,) it passes into the gasometer.

The *gasometer* or *receiver* for nitrous oxide gas is made of sheet zinc, so arranged as to receive and measure the gas, and preserve it over water. The two principal parts are the bell and tank, the bell being suspended in the tank by means of cords passing over pulleys fixed in the top of the frame work, and having weights attached to each side.

The bell is balanced by the weights; and a scale and index attached to the gasometer, registers the quantity of gas generated and also the quantity inhaled by the patient. At the base of the gasometer are two stop-cocks, one of small size to allow the gas from the purifier to enter the gasometer, and the other large, connecting with the tube through which the gas is inhaled, which tube in the interior of the gasometer opens above the level of the water.

(To be continued.)

